# Approaches to local connectivity in autism using resting state functional connectivity MRI

**DOI:** 10.3389/fnhum.2013.00605

**Published:** 2013-10-08

**Authors:** Jose O. Maximo, Christopher L. Keown, Aarti Nair, Ralph-Axel Müller

**Affiliations:** ^1^Brain Development Imaging Laboratory, Department of Psychology, San Diego State UniversitySan Diego, CA, USA; ^2^Computational Science Research Center, San Diego State UniversitySan Diego, CA, USA; ^3^Joint Doctoral Program in Clinical Psychology, San Diego State University and University of California at San DiegoSan Diego, CA, USA

**Keywords:** autism, local connectivity, functional MRI, regional homogeneity, graph theory, BOLD signal, intrinsic connectivity

## Abstract

While the literature on aberrant long-distance connectivity in autism spectrum disorder (ASD) has grown fast over the past decade, little is known about local connectivity. We used regional homogeneity and local density approaches at different spatial scales to examine local connectivity in 29 children and adolescents with ASD and 29 matched typically developing participants, using resting state functional magnetic resonance imaging data. Across a total of 12 analysis pipelines, the gross pattern of between-group findings was overall stable, with local overconnectivity in the ASD group in occipital and posterior temporal regions and underconnectivity in middle/posterior cingulate, and medial prefrontal regions. This general pattern was confirmed in secondary analyses for low-motion subsamples (*n* = 20 per group), in which time series segments with >0.25 mm head motion were censored, as well as in an analysis including global signal regression. Local overconnectivity in visual regions appears consistent with preference for local over global visual processing previously reported in ASD, whereas cingulate and medial frontal underconnectivity may relate to aberrant function within the default mode network.

## INTRODUCTION

Autism spectrum disorder (ASD) is a highly prevalent neurodevelopmental disorder (Kim et al., 2011; CDC, 2012). There is growing consensus that sensorimotor, cognitive, and sociocommunicative impairments in ASD are linked to abnormalities of functional and anatomical connectivity (Wass, 2011; Vissers et al., 2012). Evidence of aberrant white matter growth anomalies early in life (Courchesne et al., 2011), atypical white matter maturation in infants and toddlers (Weinstein et al., 2011; Wolff et al., 2012), and white matter compromise in children and adolescents with ASD (Shukla et al., 2011a) all support the relevance of connectivity as source for biomarkers of ASD. A growing body of functional connectivity magnetic resonance imaging (fcMRI) studies indeed indicates aberrant long-distance connectivity, with the predominant, though not universally replicated, finding of underconnectivity in ASD (Müller et al., 2011; Schipul et al., 2011; Vissers et al., 2012). In strange contrast, rather little is known about *short-distance* connectivity in ASD, despite evidence from postmortem studies suggesting that cytoarchitectonic abnormalities in cerebral cortex (Amaral et al., 2008), in particular the reported tight packing of cortical minicolumns with reduced lateral inhibition (Casanova and Trippe, 2009), could likely affect local connections in ASD. Theoretical arguments suggesting that local connectivity may be atypically increased in ASD (Belmonte et al., 2004; Courchesne and Pierce, 2005; Rippon et al., 2007) have been mostly speculative, although they appear consistent with some findings indicating increased cortical excitation/inhibition ratios (Rubenstein and Merzenich, 2003).

Few studies available to date have used magnetic resonance imaging (MRI) techniques to examine local connectivity, with rather divergent findings. Note, however, that the concept of “local connectivity” is not well defined and with its typically low spatial resolution, functional MRI (fMRI) detects “local” connectivity at a much coarser spatial scale than, for example, the postmortem studies of minicolumnar organization cited above (as discussed in detail in Sections “Overall Pattern of Findings and the Effect of Spatial Scale” and “Regional Patterns and Implications for the Study of Local Connectivity in ASD”). One fMRI approach uses the regional homogeneity (ReHo) approach, which implements Kendall’s coefficient of concordance (KCC) to test the homogeneity of time courses of the blood oxygen level dependent (BOLD) signal in small clusters of neighboring voxels. While originally designed for cluster purification (Zang et al., 2004), the technique has been increasingly used to examine local connectivity in a variety of clinical disorders (Dai et al., 2012; Farb et al., 2012; Yin et al., 2012; Zalesky et al., 2012; Weaver et al., 2013) as well as in the typically developing (TD) brain (Zou et al., 2009; Lopez-Larson et al., 2011; Wang et al., 2011; Anderson et al., 2013; Dong et al., 2013). Two studies have implemented ReHo in ASD. In ReHo analyses of resting state fMRI (rs-fMRI) data for 27 nearest neighboring voxels, Paakki et al. (2010) detected mixed between-group effects, with decreased ReHo in adolescents with ASD (compared to matched TD participants) in right temporal, frontal, and insular sites, accompanied by increased ReHo in right thalamus and left occipital regions. Shukla et al. (2010) used fMRI data acquired during visual search, but regressed out the modeled task effects. Nonetheless, ReHo findings for seven nearest neighbors differed heavily from those reported by Paakki et al. (2010), with increased ReHo in children and adolescents with ASD in right temporal regions, and decreased ReHo in numerous bilateral fronto-parietal sites. Notably, both of these studies used standardized ReHo (in which the KCC in each voxel is normalized by dividing it by the mean KCC), which is in principle insensitive to any potential global group differences in local connectivity. The mixed pattern of over- and underconnectivity findings in these studies was thus mandated by the analysis. However, the inconsistencies in regional patterns require further explanation, being potentially related to the finer spatial scale in the study by Shukla et al. (2010). Furthermore neither study addressed head motion, which can severely confound local BOLD correlations, at a level that can be considered adequate based on recent relevant publications (Power et al., 2012; Van Dijk et al., 2012; Satterthwaite et al., 2013; Yan et al., 2013).

The present study used a sample of rs-fMRI data that was well controlled for motion for a systematic investigation of local connectivity in ASD and matched TD adolescents, examining effects of spatial scale of local connectivity, standardization in ReHo, and the impact of head motion. A comparison analysis using the local density approach from graph theory, which has been applied to the study of local connectivity in TD adults (Sepulcre et al., 2010), was also performed.

## MATERIALS AND METHODS

### PARTICIPANTS

Magnetic resonance imaging data were collected from 37 high-functioning adolescents with ASD and 33 TD control participants. Six ASD participants with excessive head motion (as defined in Section “Motion”) were excluded from the analysis. Two further ASD and four TD participants were excluded to restore group matching on age, handedness, non-verbal IQ, and motion (see below), resulting in a final sample of 29 ASD and 29 TD participants (**Table 1**). Diagnoses in the ASD group were established using the Autism Diagnostic Interview-Revised (ADI-R; Lord et al., 1994), and the Autism Diagnostic Observation Schedule (ADOS; Lord et al., 2000). Children with ASD-related medical conditions (e.g., Fragile-X syndrome, tuberous sclerosis) or other neurological conditions (e.g., epilepsy, Tourette’s syndrome) were excluded. Participants in the TD group had no reported history of ASD or any other neurological or psychiatric condition. IQ was assessed using the Wechsler Abbreviated Scale of Intelligence–2nd edition (WASI-2; Wechsler, 1999). All participants scored above the cutoff for intellectual disability (IQ > 70). Hand preference was assessed through the Edinburgh Handedness Inventory (Oldfield, 1971). The Institutional Review Boards of San Diego State University and the University of California San Diego approved the experimental protocol. Parental informed consent was obtained for all participants, along with written assent from each participant.

**Table 1:** Demographic and diagnostic information.

	Groups		
	TD (*n* = 29)	ASD (*n* = 29)	*p*
Gender	7 female	4 female	0.31
Handedness	25 R, 4 L	26 R, 3 L	0.57
Age in years	13.5 (2.2)	13.8 (2.4)	0.74
Verbal IQ	106.2 (9.5)	105.4 (20.9)	0.84
Non-verbal IQ	108.1 (10.0)	106.3 (18.51)	0.65
Full-scale IQ	108 (8.9)	107.9 (19.0)	0.97
RMSD	0.15 (0.14)	0.14 (.11)	0.78
**ADOS algorithm score**			
Communication	–	3.4 (1.8)	–
Social reciprocity	–	8.2 (3.0)	–
Repetitive behavior	–	2.1 (1.4)	–
**ADI-R algorithm score**			
Social interaction	–	17.4 (6.2)	–
Communication	–	14.4 (6.0)	–
Repetitive behavior	–	6.3 (2.3)	–

*Values for age, IQ, and Autism Diagnostic Observation Schedule (ADOS) scores are presented as mean, with standard deviation in brackets. The *p*-value reflects group differences from χ^2^ tests (for gender and handedness) and independent *t*-tests (for all other variables). The IQ scores were missing for one individual with ASD. ADOS scores were not available for one individual, and ADI-R scores were not available for three ASD individuals. RMSD, root-mean-square of displacement; R, right; L, left.*

### MRI DATA ACQUISITION

Resting state imaging data were acquired on a GE 3T MR750 scanner with an eight-channel head coil at the University of California at San Diego Center for Functional MRI. High-resolution structural images were acquired with a standard FSPGR T1-weighted sequence (TR: 11.08 ms; TE: 4.3 ms; flip angle: 45°; FOV: 256 mm; matrix: 256 × 256; 180 slices; resolution: 1 mm^3^). Functional T2-weighted images were obtained using a single-shot gradient-recalled, echo-planar pulse sequence. One 6:10-min scan was acquired consisting of 185 whole-brain volumes (TR: 2000 ms; TE: 30 ms; slice thickness: 3.4 mm; flip angle: 90°; field of view: 220 mm; matrix: 64 × 64; in-plane resolution: 3.4 mm^2^). The first five time points were discarded to allow for T1 equilibration effects, leaving 180 time points (6 min) for analysis. Participants were instructed to keep their eyes directed on a cross-hair in the center of the projector, relax, and try not to fall asleep for the duration of the scan.

### DATA PREPROCESSING

Functional images were processed using Analysis of Functional NeuroImages software (AFNI; Cox, 1996) and FMRI software library (FSL; Smith et al., 2004). Functional images were slice-time corrected, and correction for head motion was performed by registering each functional volume to the middle time point of the scan. Field map correction was applied on each participant using in-house software for correcting magnetic resonance image distortion due to field inhomogeneity. Functional images were registered to the anatomical images via FSL’s FLIRT (Jenkinson and Smith, 2001; Jenkinson et al., 2002). Both images were resampled (3 mm isotropic) and standardized to the atlas space of the MNI152 template via FSL’s nonlinear registration tool (FNIRT) for group comparisons. In order to isolate spontaneous low-frequency BOLD fluctuations (Cordes et al., 2001), fMRI time series were bandpass filtered (0.008 < *f* < 0.08 Hz), using a second-order Butterworth filter, which was also applied to all nuisance regressors described below.

Spatial smoothing preceding ReHo statistics is an obvious critical question, because smoothness directly impacts time series correlations between neighboring voxels (Zuo et al., 2013). Our primary preprocessing pipeline therefore did not include a smoothing step. However, spatial smoothness may differ across data sets due to varying interpolation associated with motion correction and spatial normalization. In order to minimize effects of varying smoothness, a secondary analysis setting the effective smoothness of all data sets to a Gaussian FWHM of 6 mm, using AFNI’s 3dBlurToFWHM, was performed. Results are presented in **Figure 2D**. For density analyses, we followed the preprocessing pipeline by Sepulcre et al. (2010), which included smoothing with a Gaussian kernel (FWHM 6 mm). Linear effects attributable to scanner drift were removed during regression.

Six rigid-body motion parameters acquired from motion correction and their derivatives were regressed from the images. In order to remove signal from cerebral white matter and lateral ventricles, masks were created at the participant level, using FSL’s FAST automated segmentation (Zhang et al., 2001). Masks were trimmed to avoid partial-volume effects, and an average time series for each region was extracted and removed via regression. Derivatives for white matter and ventricular time series were also computed and removed, for a total of 16 nuisance regressors. All main analyses were performed without global signal regression (GSR) to avoid the creation of spurious anti-correlations (Murphy et al., 2009), which may substantially distort group differences (Saad et al., 2012). Nonetheless, an additional ReHo analysis including GSR was performed using a cluster size of 27 voxels. Results are presented in **Figure 2E**.

### MOTION

Motion was quantified as the Euclidean distance calculated from the six rigid-body motion parameters for two consecutive time points. For any instance >1.0 mm, considered excessive motion, the time point as well as the immediately preceding and subsequent time points were censored, or “scrubbed” (Power et al., 2012). If two censored time points occurred within 10 time points of each other, all time points between them were also censored. Participants with fewer than 80% of time points remaining after censoring were excluded from the analysis. The two groups did not significantly differ in the number of retained time points (*M* = 177 in each group, *p* = 0.94). Average head motion over each participant’s session was defined as the root mean square of displacement (RMSD) and did not significantly differ between groups (*p* = 0.78). For more detailed analysis of head motion, a two-way analysis of variance (ANOVA) was conducted to test the effects of group and type of motion (three translational and three rotational). The interaction of group and motion type was not significant, *F*(5,342) = 0.307, *p* = 0.91. Additionally, we correlated KCC from ReHo27, averaged across all brain voxels, for cluster size 27 with RMSD values to determine the relationship between connectivity and motion. There was no significant correlation between these two measures, *r* = -0.135, *p* = 0.54.

For further protection against potential effects of head motion on local connectivity measures, a low-motion subsample was identified and a more conservative censoring threshold of >0.25 mm was applied. Participants who had less than 80% of their time points remaining after censoring were excluded from both analysis. Both groups were matched for gender, handedness, age, verbal IQ, non-verbal IQ, full-scale IQ, and motion (**Table 2**). The final low-motion subsample consisted of 42 participants (TD = 22; ASD = 20).

**Table 2:** Demographic and d2iagnostic information for low-motion subsamples.

	Groups		
	TD (*n* = 22)	ASD (*n* = 20)	*p*
Gender	4 female	2 female	0.44
Handedness	18 R, 4 L	14 R, 3 L	0.97
Age in years	14.0 (1.82)	14.37 (2.07)	0.65
Verbal IQ	107.5 (9.76)	110.79 (20.37)	0.50
Non-verbal IQ	107.95 (10.39)	109.32 (18.74)	0.77
Full-Scale IQ	108.68 (9.46)	110.45 (19.93)	0.71
RMSD	0.05 (0.02)	0.05 (0.03)	0.84
**ADOS algorithm score**			
Communication	–	3.1 (1.9)	–
Social reciprocity	–	7.8 (2.8)	–
Repetitive behavior	–	1.8 (1.2)	–
**ADI-R algorithm score**			
Social interaction	–	16.8 (6.5)	–
Communication	–	13.8 (6.0)	–
Repetitive behavior	–	6.2 (2.4)	–

*Values for age, IQ, and Autism Diagnostic Observation Schedule (ADOS) scores are presented as mean and standard deviation (SD). The *p*-value is from χ^2^ tests and independent *t*-tests for differences between groups. Handedness scores were missing for three ASD participants, IQ scores were missing for one ASD participant, and ADI-R scores were not available for another ASD participant. RMSD, root-mean-square difference; R, right; L, left.
*

### LOCAL FUNCTIONAL CONNECTIVITY MEASURES

#### Regional homogeneity

Regional homogeneity implements KCC, which relies on rank correlations of time series to assess the homogeneity of a given center voxel and its neighboring voxels. KCC within a given cluster of voxels is equal to the parameter *W* (ranging from 0 to 1)

$$W=\frac{\Sigma {\left(R_{i}\right)}^{2}-{n(\overset{¯}{R})}^{2}}{\frac{1}{12}K^{2}\left(n^{3}-n\right)},$$

where *R*_i_ is the sum rank of the *i*th time point; $\overset{¯}{R}$ is the mean of the *R*_i_s; *K* is the number of time series within a selected cluster (7, 19, or 27 voxels), and *n* is the number of ranks, as determined by the number of time points (Zang et al., 2004).

For this study, ReHo was computed for cluster sizes of 7, 19, and 27 voxels (abbreviated “ReHo7,” “ReHo19,” and “ReHo27,” respectively), which correspond to the smallest cluster (a reference voxel and its six immediate neighbors) and small gradual symmetric expansions of this cluster. In order to further examine spatial scale effects, ReHo was also computed using a radius of 14 mm (407 voxels; “ReHo14mm”), corresponding to a radius used in the density analysis (described below). A gray-matter mask was used to avoid partial-volume effects. All individual ReHo maps were obtained using AFNI’s 3dReHo command. Individual voxel-wise ReHo maps were standardized into KCC–ReHo *z*-values by subtracting the mean voxel-wise KCC–ReHo obtained for the entire whole-brain mask (i.e., global KCC–ReHo), and then dividing by the standard deviation. An additional analysis without standardization was performed (for ReHo27 only) to detect any potential global group differences in local connectivity. All ReHo maps were smoothed to a Gaussian FWHM of 6 mm for better anatomical comparability of ReHo values on the group level, using AFNI’s 3dBlurToFWHM. Group differences were examined with two-sample *t*-tests (3dttest). To correct for multiple comparisons, Monte Carlo simulations via AFNI’s 3dClustSim command were applied to obtain a corrected significance level of *p* < 0.05 (using a voxelwise threshold of *p* < 0.05, uncorrected, and a minimum cluster size of 55 voxels).

**FIGURE 1:**
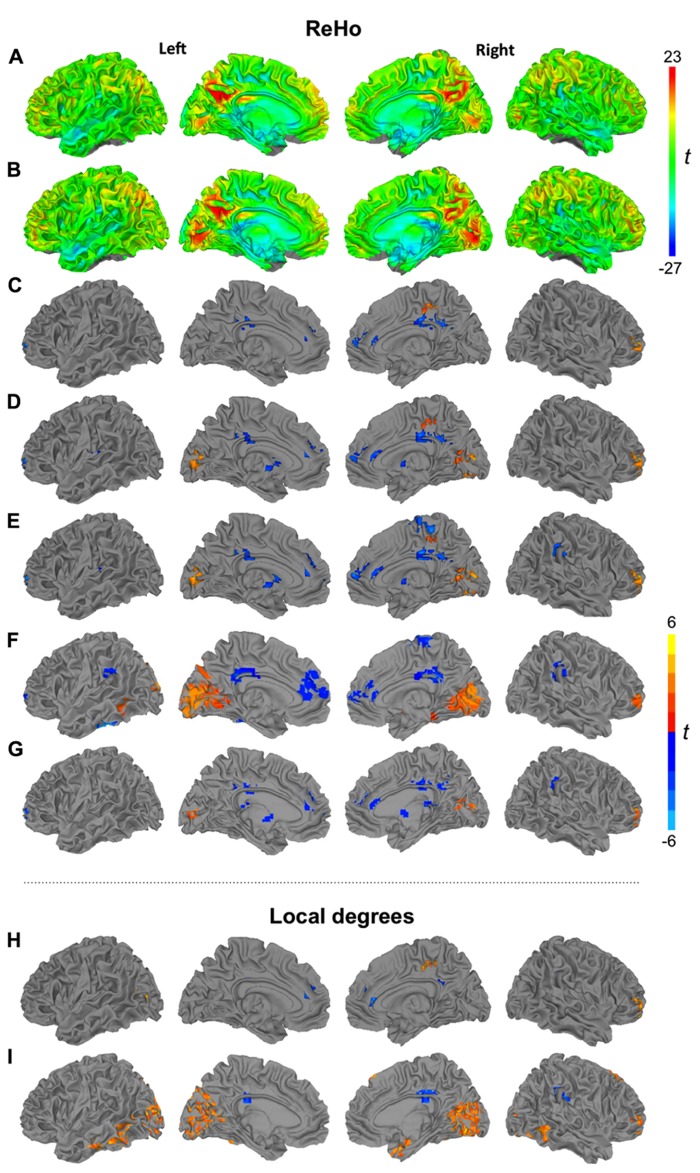
Surface renderings of ReHo (27 voxels) for TD **(A)** and ASD groups **(B)**. Clusters of significant group differences between TD and ASD groups in ReHo using cluster sizes of 7 **(C)**, 19 **(D)**, 27 voxels **(E)**, 14 mm radius **(F)**, and non-standardized ReHo (27 voxels) **(G)**. Significant group differences between TD and ASD groups of density analysis using radii of 6 **(H)** and 14 mm **(I)**. All clusters *p* < 0.05, corr. warm colors: ASD > TD; cool colors: TD > ASD.

The relationship between local connectivity and symptom severity was further examined focusing on regions with significant group differences. Two separate combined clusters were created (all clusters of overconnectivity and all clusters of underconnectivity) based on group comparison for ReHo27 (yellow and blue clusters in **Figure 1E**, respectively) and Pearson’s correlation analyses were performed between *z*-scores from KCC (averaged across all voxels within combined over- and underconnectivity clusters, respectively) and ADOS, and ADI scores (as listed in **Table 1**).

#### Density analysis

Local functional connectivity was further examined using connection density, as previously applied in neurotypical adults (Sepulcre et al., 2010). In graph theory, connection “density” is defined as the number of “edges” (connections) of a “node” (here: voxel) in proportion to the total number of possible edges (Bullmore and Sporns, 2009). We implemented this measure by calculating the “degree” of each voxel, i.e., the number of neighboring voxels with BOLD time series correlation at *r* > 0.25 (*p* < 0.001) within a 6 and 14 mm radius from the reference voxel, based on the Euclidean distance between the centers of voxel pairs. A 14 mm radius allowed comparison of results with those reported by Sepulcre et al. (2010) who used the same radius, whereas an additional 6 mm radius was chosen for comparison with ReHo27. To generate a connectivity map for each group, local degrees were converted to *z*-scores. Group comparisons were performed using two-sample *t*-tests. Multiple comparison correction was performed as described above.

## RESULTS

### ReHo

We first inspected ReHo within each group for the most commonly used cluster size of 27 voxels (**Figures 1A, B**). Patterns were highly similar for TD and ASD groups, with bilateral hotspots in posterior cingulate gyrus extending into precuneus. A hotspot in the ASD group in striate and extrastriate cortex appeared less pronounced in the TD group. Further regions of relatively high ReHo were seen in superior parietal, frontopolar, and medial frontal regions.

Despite the overall similar patterns on within-group maps, localized group differences were detected for ReHo at all four spatial scales (7, 19, 27 voxels, 14 mm radius; **Figures 1C–F**; **Table 3**). All of these analyses showed underconnectivity in the ASD group compared to the TD group in left superior frontal gyrus and bilateral cingulate cortex, accompanied by overconnectivity in right middle frontal gyrus. Generally, between-group effects were more modest at the finer spatial scales, and additional clusters of under- and overconnectivity were detected with increasingly coarse spatial scale. For example, underconnectivity in the ASD group was detected in right paracentral regions only for ReHo27 and ReHo14mm. Extensive overconnectivity effects were detected in bilateral striate and extrastriate cortices at 14 mm radius, which were smaller for ReHo27 and ReHo19, and absent for ReHo7. Overconnectivity effects in parahippocampal, temporal, and supramarginal regions were also only detected at the coarsest spatial scale (14 mm radius). Non-standardized ReHo27 (**Figure 1G**) yielded very similar group differences compared to standardized ReHo at the same spatial scale.

**Table 3:** Clusters of significant group differences in ReHo.

ReHo cluster size (voxels)	Region	Cluster volume (μl)	Peak coordinates			Peak
			*x*	*Y*	*z*	*t*
7	TD > ASD					
	Right precuneus	3888	14.5	-50.5	33.5	-3.8
	Left superior frontal gyrus	1944	-18.5	60.5	3.5	-5.0
	Left anterior cingulate cortex	1917	-0.5	45.5	18.5	-3.9
	ASD > TD					
	Right middle cingulate cortex	2349	17.5	-20.5	48.5	4.5
	Right middle frontal gyrus	1809	41.5	54.5	6.5	5.0
19	TD > ASD					
	Left middle cingulate cortex	4941	-0.5	-29.5	33.5	-4.2
	Right anterior cingulate cortex	3456	2.5	39.5	9.5	-4.2
	Left putamen	2322	-27.5	-14.5	12.5	-3.8
	Left superior frontal gyrus	2025	-18.5	60.5	3.5	-4.6
	Left caudate	1917	-6.5	12.5	6.5	-4.7
	ASD > TD					
	Left calcarine gyrus	3753	-3.5	-86.5	3.5	3.2
	Right middle frontal gyrus	3267	41.5	54.5	6.5	4.9
	Right paracentral lobule	2403	17.5	-35.5	48.5	4.1
	Right fusiform gyrus	2295	32.5	-68.5	-8.5	3.6
27	TD > ASD					
	Left middle cingulate cortex	5400	-0.5	-29.5	33.5	-4.2
	Right superior medial gyrus	4077	8.5	57.5	9.5	-4.1
	Left putamen	2349	-27.5	-14.5	12.5	-3.3
	Right paracentral lobule	2106	2.5	-32.5	60.5	-3.1
	Left superior frontal gyrus	2079	-18.5	60.5	3.5	-4.7
	Left caudate	2052	-6.5	12.5	6.5	-4.8
	Right supramarginal gyrus	1809	65.5	-38.5	30.5	-3.3
	ASD > TD					
	Left calcarine gyrus	4239	-3.5	-86.5	3.5	3.2604
	Right middle frontal gyrus	3240	41.5	54.5	6.5	4.5738
	Right fusiform gyrus	2997	32.5	-68.5	-8.5	3.3385
	Right middle cingulate gyrus	1971	20.5	-32.5	51.5	3.8254
27 (Non-standardized)	TD > ASD					
	Right middle cingulate cortex	4536	2.5	-23.5	33.5	-3.8
	Right anterior cingulate cortex	2862	2.5	39.5	9.5	-3.5
	Left caudate	2484	-6.5	9.5	6.5	-4.5
	Left superior frontal gyrus	2052	-18.5	60.5	3.5	-4.3
	Right supramarginal gyrus	1809	65.5	-38.5	30.5	-2.9
	Left thalamus	1566	-3.5	-29.5	12.5	-3.0
	ASD > TD					
	Right calcarine gyrus	2268	5.5	-68.5	12.5	2.8
	Right middle frontal gyrus	2133	41.5	54.5	6.5	4.2
407 (14 mm)	TD > ASD					
	Left superior medial gyrus	5265	-3.5	42.5	27.5	-2.9
	Left cingulate gyrus	4131	-3.5	-26.5	27.5	-3.7
	Left inferior temporal gyrus	3348	-51.5	-35.5	-26.5	-3.4
	Right supramarginal gyrus	1836	65.5	-35.5	27.5	-3.7
	Right anterior cingulate cortex	1755	2.5	42.5	9.5	-2.9
	Left supramarginal gyrus	1701	-57.5	-38.5	27.5	-2.8
	Right superior medial gyrus	1215	2.5	54.5	9.5	-2.9
	ASD > TD					
	Left lingual gyrus	29808	-15.5	-68.5	3.5	4.3
	Right parahippocampal gyrus	3051	29.5	-29.5	-17.5	3.1
	Right middle frontal gyrus	2511	44.5	54.5	3.5	3.5
	Left middle temporal gyrus	1620	-66.5	-47.5	-8.5	2.8
	Right supp. motor area	1161	5.5	-14.5	75.5	-3.0

Local connectivity from ReHo27 was positively correlated with ADI-R communicative scores in clusters of underconnectivity in the ASD group (all blue clusters in **Figure 1E** combined), *r* = 0.43, *p* = 0.04, as well as in clusters of overconnectivity (all yellow clusters in **Figure 1E** combined), *r* = 0.48, *p* = 0.02. There were no significant correlations with ADOS scores. However, note that these analyses were performed for exploratory purposes and caution is required given that no correction for multiple comparisons was performed.

### DENSITY ANALYSIS

The patterns of group differences for local degrees (density of connections) were overall similar to the ReHo findings (**Figures 1H, I**; **Table 4**). Comparing only two spatial scales (radii of 6 mm, corresponding to ReHo27, and 14 mm), we again found much more robust between-group effects for the coarser spatial scale. However, there was less regional consistency: only a single effect – local overconnectivity in right middle frontal gyrus in the ASD group – was found in both analyses. Overconnectivity in right medial paracentral cortex and underconnectivity in bilateral anterior cingulate cortex were detected only for a 6 mm radius. Conversely, extensive overconnectivity clusters in bilateral striate and extrastriate as well as right temporopolar cortices were only detected at a 14 mm radius, as was underconnectivity in middle/posterior cingulate gyri bilaterally.

**Table 4:** Clusters of significant group differences in density analysis.

Radius (mm)	Region	Cluster volume (μl)	Peak coordinates			Peak
			*x*	*y*	*z*	*t*
6	TD > ASD					
	Right posterior cingulate cortex	2133	2.5	-41.5	24.5	-4.1
	Left anterior cingulate cortex	1701	-3.5	42.5	21.5	-4.1
	ASD > TD					
	Right middle frontal gyrus	2376	41.5	51.5	6.5	4.3
	Right postcentral gyrus	2025	23.5	-26.5	48.5	4.0
	Left middle temporal gyrus	1485	-54.5	-65.5	12.5	3.5
14	TD > ASD					
	Right supramarginal gyrus	1809	-65.5	32.5	24.5	-3.7
	Right middle cingulate cortex	1512	-2.5	29.5	33.5	-3.6
	ASD > TD					
	Right cuneus	41013	5.5	-74.5	18.5	4.5
	Left inferior temporal gyrus	9126	-48.5	-47.5	-23.5	4.1
	Right inferior temporal gyrus	3915	53.5	-53.5	-8.5	4.1
	Left cuneus	2565	-9.5	-92.5	30.5	3.4
	Right superior medial gyrus	2349	5.5	33.5	57.5	4.0
	Right middle orbital gyrus	2187	35.5	54.5	-2.5	3.4
	Right middle temporal gyrus	1755	44.5	6.5	-26.5	3.0
	Right parahippocampal gyrus	1620	23.5	6.5	-29.5	3.9

### SECONDARY ANALYSIS IN LOW-MOTION SUBSAMPLE

#### ReHo

**FIGURE 2:**
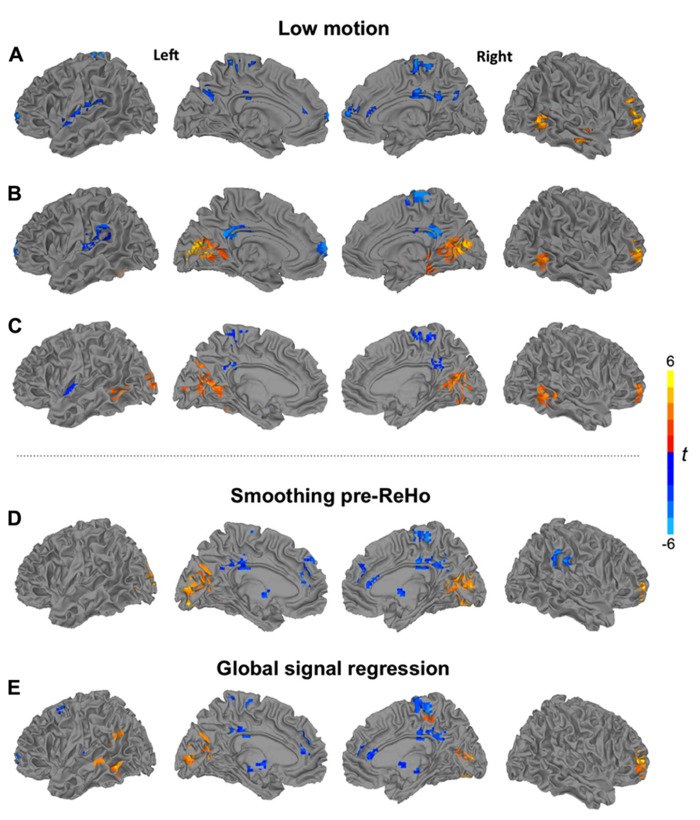
Clusters of significant group differences between low-motion TD and ASD groups for ReHo using **(A)** a cluster size of 27 voxels, and **(B)** a radius of 14 mm, and **(C)** for 14 mm density analysis. Analyses of ReHo (27 voxels) showing clusters of significant group differences **(D)** for pipeline applying spatial smoothing before ReHo statistics and **(E)** for pipeline including global signal regression. All clusters *p* < 0.05, corr; warm colors: ASD > TD; cool colors: TD > ASD.

Additional analyses using ReHo27 (**Figure 2A**) and ReHo14mm (**Figure 2B**) were performed for the low-motion subsample. For both scales, underconnectivity in the ASD group was found in left perisylvian and frontopolar regions, as well as in bilateral middle/posterior cingulate gyrus and right paracentral cortex, accompanied by overconnectivity in right middle frontal and middle temporal gyri. Extensive overconnectivity in visual regions around the calcarine fissure was only seen at the 14 mm radius.

#### Density analysis

For a 14 mm radius (**Figure 2C**), results were mostly consistent with the corresponding ReHo analysis and the corresponding density analysis for the full sample. Widespread overconnectivity was detected in bilateral occipital and posterior temporal regions, as well as right middle frontal gyrus, while underconnectivity was found in left insula, bilateral precuneus, and middle/posterior cingulate gyrus.

## DISCUSSION

### OVERALL PATTERN OF FINDINGS AND THE EFFECT OF SPATIAL SCALE

Across 12 different analysis pipelines (**Figures 1C–I** and 2A–E), regional patterns of between-group differences were overall stable, with increased local connectivity in the ASD group detected in bilateral striate and extrastriate as well as right lateral prefrontal cortices, accompanied by reduced local connectivity in anterior and posterior cingulate and medial prefrontal regions. We generally observed a trend toward more robust between-group findings for coarser spatial scales, corresponding to radii of ≥6 mm. This may be primarily attributed to sampling from a larger number of voxels, which probably improved the signal-to-noise ratio. Effects that were seen at lower, but not at higher spatial scales, such as overconnectivity in ASD in right inferior paracentral regions (**Figures 1C– E** vs. **Figure 1F**) are therefore noteworthy, as they might reflect group differences occurring only at the more local levels. Some effects, such as underconnectivity in anterior and posterior cingulate gyrus as well as medial prefrontal cortex, were also remarkably stable across radii from c. 3 to 14 mm. On the other hand, effects in striate and extrastriate visual cortices were much more robust at higher spatial scales and in fact not at all detected in ReHo7 analysis (for interpretation of regional patterns, see Regional Patterns and Implications for the Study of “Local Connectivity” in ASD).

While these findings may suggest an overall superiority of analyses at coarser spatial scales (when assessed on the basis of robustness of between-group effects), any such conclusion very much depends on the exact goals of the investigation. The study of local connectivity by fMRI is generally hampered by this technique’s typically modest spatial resolution, which limits its sensitivity to abnormalities of local cortical organization suspected in ASD. For example, smaller size and increased density of cortical minicolumns with reduced lateral inhibition has been reported in postmortem studies from one group (Casanova et al., 2006; Casanova and Trippe, 2009). As minicolumns measure c. 30–50 μm in width, the spatial resolution in our study was too low by at least a factor of 100 to capture individual minicolumns. Although it is possible that basic abnormalities of minicolumnar organization, such as the suspected reduced lateral inhibition, could affect the BOLD signal and its local correlations at the resolution common in fMRI, this remains speculative. Widening the spatial scale, beyond what is dictated by raw image resolution (3.4 mm in this study), may therefore yield cleaner or more robust results; however, these probably reflect abnormalities at a different level of complexity, compared to those described in the postmortem literature. Nonetheless, it is likely that anatomical parameters, such as cortical thickness, may have some impact on measures of local functional connectivity. Given the mentioned relatively low spatial resolution of fMRI data, some voxels in a ReHo cluster, for example, may show partial volume effects (part of the voxel falling onto the gray/white boundary), which would be expected to reduce BOLD correlations with a neighboring reference voxel in pure gray matter. Resulting reductions in ReHo will most likely occur to a lesser extent in cortex with greater thickness. However, investigation of such links was beyond the scope of the current study. The described issue here solely serves to illustrate the intimate links between local functional connectivity and local cortical anatomy.

Density analyses showed a similar pattern of between-group effects compared to ReHo analyses, although underconnectivity clusters were overall less robust and one overconnectivity cluster in right anterior mediotemporal cortex was seen only in the density analysis for a 14 mm radius (but not in ReHo14mm). This may relate to methodological differences: Whereas ReHo is based on rank ordering of time series to assess the homogeneity in voxels within a cluster of chosen size, density reflects degrees, i.e., the number of voxels exceeding a threshold Pearson’s correlation (here, *r* > 0.25). ReHo is therefore more sensitive to the strength of correlations within a cluster of selected volume (e.g., 7 or 27 voxels), whereas in our density analysis, weak correlations were discarded and local connectivity was solely assessed with respect to the number (not the strength) of the connections exceeding the correlation threshold. Differences in sensitivity are therefore not unexpected.

### MOTION, SMOOTHNESS, GLOBAL SIGNAL, AND ReHo STANDARDIZATION

Recent investigations have highlighted the significant impact of even small amounts of head motion on fcMRI measures (Power et al., 2012; Van Dijk et al., 2012; Satterthwaite et al., 2013; Yan et al., 2013). We therefore also ran analyses in a low-motion subsample, including only the 42 participants with >80% time points remaining after applying a more conservative censoring threshold of 0.25 mm. The pattern of findings for this subsample was overall similar to the one seen in the full sample, suggesting that our results were well protected against motion confounds.

Image smoothness is of particular importance in *local* connectivity analyses because smoothing by definition inflates the correlation between neighboring voxels. In order to avoid this issue, our primary analysis pipeline implemented smoothing only subsequent to ReHo statistics. However, even without an explicit smoothing step, preprocessing requires intermodal alignment (of functional to high-resolution structural volumes), motion correction (alignment across time points), and spatial normalization, which unavoidably increases image smoothness (even when intermodal alignment and spatial normalization are performed in a single interpolation, as in our study). Smoothness may therefore vary across individual datasets, and more importantly, across groups, potentially confounding comparisons of local connectivity. We therefore performed an additional ReHo27 analysis, setting the smoothness of all datasets to an effective Gaussian FWHM of 6 mm. This analysis (**Figure 2D**) yielded almost identical results to the primary analysis without pre-statistic smoothing (**Figure 1C**), indicating that differences in image smoothness did not confound our group comparisons.

We further considered the question of GSR, the pros and cons of which have been debated for several years in the fcMRI literature. Arguments against the procedure include findings that at least some components of global signal fluctuations likely reflect true neuronal activity (Schölvinck et al., 2010) and that GSR may induce spurious negative correlations of BOLD time series (Murphy et al., 2009). Nonetheless, GSR has been found highly effective in removing noise, especially reducing the effects of head motion on BOLD correlations (Yan et al., 2013). We therefore included GSR in one analysis (ReHo27) and found that removal of the global signal had some effect (e.g., highlighting overconnectivity effects in the ASD group in left lateral temporal cortex not seen in ReHo27 without GSR), but did not dramatically change the overall pattern of findings. Thus, while GSR may in principle bear the risk of distorting fcMRI group comparisons (Saad et al., 2012), this was not the case in our data set.

A final methodological issue concerned ReHo standardization. As initially advocated by Zang et al. (2004), conversion of KCC (W) into *z*-maps was performed in both previous ReHo studies of ASD (Paakki et al., 2010; Shukla et al., 2010). This standardization mandates a distribution of whole brain ReHo maps around zero. While advantageous for teasing out regionally specific differences in ReHo, this procedure bears the risk of type II error in group comparisons, if there are global differences in ReHo between groups (because the whole brain mean ReHo in each individual participant from both groups is equally set to zero). It is therefore possible in principle that the mixed pattern of effects seen on our ReHo analyses (with both clusters of overconnectivity and underconnectivity) could be due to the standardization step – a possibility not considered in previous ASD ReHo studies. However, non-standardized ReHo27 (i.e., without conversion of KCC to *z*) yielded highly similar results to standardized ReHo27 (**Figure 1G** vs. **Figure 1E**). Overall subtly more robust between-group effects for standardized ReHo can be attributed to lower variance due to *z*-conversion. It is therefore unlikely that the mixed pattern of over- and underconnectivity findings in our study was an artifact of ReHo standardization.

### COMPARISON WITH PREVIOUS STUDIES

As noted, the two previous ReHo studies of ASD by Paakki et al. (2010) and Shukla et al. (2010) reported highly divergent findings. The convergence between either of them and the current study was equally modest, with only a few roughly consistent findings. These included local overconnectivity in ASD in occipital lobe (also detected by Paakki et al., 2010) and underconnectivity in ASD in left superior frontal gyrus, precuneus, and posterior cingulate gyrus, and overconnectivity in right fusiform gyrus (also observed by Shukla et al., 2010). While differences between our study and the study by Shukla et al. (2010) may be attributed to the use of resting vs. task-activated fMRI data, respectively, the even more pronounced inconsistencies with the study by Paakki et al. (2010) may appear less transparent at first, given that these authors also used resting state data. Participants were probably overall older in the study by Paakki et al. (2010), although no exact demographic data are provided and matching on important variables, such as age, sex, handedness, and non-verbal IQ, is either not mentioned or not available due to lack of data (e.g., IQ). It is therefore hard to determine whether demographic factors may have affected the pattern of results in the study by Paakki et al. (2010).

There were also differences in imaging methods. While acquisition at lower spatial resolution (70.4 vs. 39.3 μl voxels) and at lower field strength (1.5 vs. 3T) in the study by Paakki et al. (2010), compared to ours, may have had some effect, the differential treatment of head motion (and other noise components) is probably crucial in explaining differential findings. Paakki et al. (2010) performed solely conventional motion correction. Noise components removed through regression of six translational and rotational motion times series, time series from white matter and ventricles (and their derivatives) in our study were thus retained in this earlier study. In view of recent methods investigations (Power et al., 2012; Van Dijk et al., 2012; Satterthwaite et al., 2013; Yan et al., 2013), which were not available to Paakki et al. (2010) and which highlight the exquisite sensitivity of fcMRI (including local connectivity and ReHo) analyses to even small amounts of motion, the confidence in these earlier findings therefore has to be low. Furthermore, no group matching for head motion or censoring (“scrubbing”) of motion-affected time points was reported by Paakki et al. (2010). Conversely, the study by Shukla et al. (2010) performed censoring (albeit at an all-too liberal threshold of 2 mm) and ascertained approximate group matching for motion (*p* = 0.7 for translations; *p* = 0.3 for rotations). In this context, the relatively greater (though still modest) consistency of findings between this study and the present one may be noted.

### REGIONAL PATTERNS AND IMPLICATIONS FOR THE STUDY OF “LOCAL CONNECTIVITY” IN ASD

As already alluded to above, the term “local connectivity” is ill-defined, encompassing spatial scales from a few microns to millimeters and even centimeters. While there has been some indirectly supporting evidence (Rubenstein and Merzenich, 2003; Casanova and Trippe, 2009), the theoretical idea of atypically increased local connectivity in ASD (Belmonte et al., 2004; Courchesne and Pierce, 2005; Rippon et al., 2007) therefore requires specification of scale. The expectation that fMRI, using ReHo or local density techniques, or diffusion tensor imaging (Shukla et al., 2011b) may provide empirical tests of the local overconnectivity hypothesis applies at best to the coarsest spatial scales included under the vague umbrella term of “local connectivity.” At these relative coarse scales, the mixed pattern of our findings (with regions of both atypically increased and decreased connectivity) does not support *general* local overconnectivity in ASD. This compares with a study by Anderson et al. (2011) who found no general overconnectivity for connections at a distance below 25 mm in adolescents and adults with ASD. As measures were collapsed across the whole brain in this latter study, such non-finding can be reconciled with regionally specific effects in both directions (increased and reduced), as detected in our study. Note that the conclusion in the study by Anderson et al. (2011) of short-distance connections not being strongly informative for machine learning classification (ASD vs. TD) may be due to the mentioned whole brain approach and does not rule out predictive power for region-specific local connectivity patterns.

The finding of robust and extensive overconnectivity in striate and extrastriate visual cortex, at scales above 6 mm, is intriguing in view of potential local biases in visual perception, supported thus far mainly by findings from behavioral studies (Dakin and Frith, 2005; Mottron et al., 2006). Unusual profiles of visual perception have been observed in many studies (as reviewed in Simmons et al., 2009). Remarkable are islets of superior abilities in visual search (O’Riordan, 2004), associated with increased functional connectivity during visual search performance (Keehn et al., 2012). In a meta-analysis, Samson et al. (2012) found overall greater activity in ASD groups compared to TD control groups in posterior brain regions for a variety of visual processing tasks (from studies using face, object, and word stimuli), including occipitotemporal regions, for which local overconnectivity was detected in the present study. Greater activation and increased local connectivity could be directly related, as increased spontaneous BOLD signal correlations in visual cortex (as detected in our ReHo and density analyses) may also enhance BOLD signal changes in response to a task (as in the studies reviewed by Samson et al., 2012). Indeed, effects of resting state signal fluctuations on amplitude of stimulus-induced response have been observed in a number of fMRI and electrophysiological studies (as reviewed in Northoff et al., 2010). Specifically with respect to visual cortex, Liu et al. (2011) reported that local BOLD correlations were positively associated with amplitude of response to simple visual stimuli.

Conversely, regions that consistently (across different analysis pipelines) showed local underconnectivity in ASD included posterior cingulate cortex and medial prefrontal lobe. Both of these belong to a system that has been found active during the resting state and is considered a “default mode network” (DMN; Raichle et al., 2001). Several fcMRI studies have examined the DMN in ASD, with the overall consistent finding of reduced connectivity between DMN nodes, such as posterior cingulate and medial prefrontal cortices (Monk et al., 2009; Assaf et al., 2010; von dem Hagen et al., 2012). The present findings suggest that such reduced long-distance connectivity between regions of the DMN is accompanied by local underconnectivity *within* these regions. This is consistent with a recent finding by Lynch et al. (2013) who observed underconnectivity in children with ASD between posterior cingulate gyrus and neighboring regions (precuneus, retrosplenial cortex). Notably, this latter study as well as the one by Monk et al. (2009) found that underconnectivity within the DMN was accompanied by atypically increased functional connectivity of DMN regions with regions outside this network, such as medial and lateral temporal cortices. Our finding may also be consistent with atypically reduced connectivity in bilateral precuneus observed by Di Martino et al. (2013) in children with ASD. Note, however, that this latter finding compares only indirectly to ours, as Di Martino et al. (2013) tested degree centrality, which is a graph theory construct reflecting both short- and long-distance connectivity of each node.

A further finding that was overall stable across analysis pipelines was an asymmetry of reduced connectivity in left, but increased connectivity in right anterior prefrontal regions. The asymmetric effects in anterior prefrontal cortex may be related to a recent finding of right-hemisphere shifts of functional networks in ASD (Cardinale et al., 2013). Expanding on a few previous studies (e.g., Boddaert et al., 2003; Eyler et al., 2012) that had suggested greater right-hemisphere participation in language-related processing in ASD, the study by Cardinale et al. (2013) indicated that such right-hemisphere shifts may be a pervasive feature of functional brain organization in ASD, applying to many functional networks, including non-verbal ones and those with participation of anterior prefrontal cortices. Such rightward shifts may be associated by atypically increased local connectivity in right compared to left prefrontal cortex in ASD, as observed in the pattern of group differences detected in our study. However, such potential links need to be considered with caution, given that this asymmetric pattern of group differences was solely found in anterior prefrontal cortex, whereas the rightward shifts observed by Cardinale et al. (2013) occurred in widely distributed functional networks.

Local connectivity abnormalities, as detected in our study, may relate to recent evidence suggesting reduced functional differentiation of cerebral cortex. Shih et al. (2011) first reported such reduced functional differentiation in posterior superior temporal sulcus in children and adolescents with ASD. Neighboring subregions were found to be less differentiated, both in the temporal domain (with respect to BOLD time series) and in the spatial domain (with respect to whole brain connectivity patterns). Analogous findings for primary motor cortex have been reported by Nebel et al. (2012) who observed that functional differentiation between lower limb and trunk regions vs. upper limb and hand regions was reduced in children with ASD. Findings from these two studies may be consistent with a general model of reduced network segregation in ASD, as proposed in two studies by Rudie et al. (2012, 2013). Reduced network segregation, accompanied by impaired local functional differentiation, may relate to findings of regional local overconnectivity. For example, reduced differentiation in posterior superior temporal sulcus, as reported by Shih et al. (2011), is equivalent to atypically increased correlations in neighboring voxels, and thus corresponds to local overconnectivity in ASD in this region, as detected by us in analyses at coarser spatial scales (**Figures 1F**, I). On the other hand, local underconnectivity could reflect reduced integration within specialized functional networks, such as the DMN, as discussed above. However, these potential links across studies remain speculative, as long as direct comparisons of local connectivity and network segregation in the same cohort are unavailable.

Our results also compare in interesting ways to a recent magnetoencephalography (MEG) study by Khan et al. (2013), who reported reduced local functional connectivity within the fusiform face area in response to face and house stimuli in adolescents with ASD. This may at first glance appear at odds with our findings of overconnectivity in fusiform gyrus in some of the analyses. However, note that the cited MEG study operationalized “local connectivity” by testing the phase–amplitude coupling between alpha and gamma bands, which presumably reflects *inhibitory* connectivity. This differs fundamentally from physiological mechanisms boosting correlations of the BOLD signal, which likely rely primarily on excitatory connectivity. Ours and the findings from Khan et al. (2013) may thus well be compatible, indicating increased excitatory and reduced inhibitory local connectivity in inferior occipitotemporal regions, respectively.

### CHALLENGES AND CONCLUSIONS

We found that local connectivity was atypical in adolescents with ASD, with overconnectivity – mostly in occipital and posterior temporal regions – accompanied by underconnectivity in cingulate and medial frontal sites. While the consistency of findings across different analysis pipelines and in low-motion subsamples was reassuring, many challenges remain for a full understanding of local connectivity, both at the methodological and conceptual levels. Methodologically, limits of spatial and temporal resolution may best be approached in future studies combining hemodynamic and electrophysiological techniques. However, a spatial resolution adequate for the *in vivo* study of cytoarchitectonic anomalies, which may affect local connectivity in ASD, is unlikely in the foreseeable future and there is a need for mechanistic models that will allow a prediction of effects that can be detected with fMRI, based on post mortem findings on cellular organization.

Although only a first step in this direction, our findings indicate that local connectivity at a relatively coarse spatial scale is aberrant in ASD. However, the patterns of these aberrations were inconsistent with previous simple hypotheses about “local overconnectivity contrasting with long-distance underconnectivity” (as described in the Introduction), suggesting instead regionally specific abnormalities of local connectivity, whose functional significance (e.g., in the visual system) is only beginning to emerge.

## Conflict of Interest Statement

The authors declare that the research was conducted in the absence of any commercial or financial relationships that could be construed as a potential conflict of interest.
